# The genus *Omphreus* in Bosnia and Herzegovina and Montenegro, with two new subspecies of *O. morio* (Coleoptera, Carabidae, Omphreini)

**DOI:** 10.3897/zookeys.509.9506

**Published:** 2015-06-25

**Authors:** Srećko Ćurčić, Riccardo Sciaky, Dragan Antić, Nikola Vesović

**Affiliations:** 1 Institute of Zoology, University of Belgrade - Faculty of Biology, Studentski Trg 16, 11000 Belgrade, Serbia; 2 Via Fiamma 13, 20129 Milan, Italy

**Keywords:** Ground beetles, soil-dwelling fauna, Balkan Peninsula

## Abstract

Two new ground beetle subspecies, Omphreus (Omphreus) morio sandeli **ssp. n.** (from Mts. Zelengora and Maglić, eastern Bosnia and Herzegovina) and Omphreus (Omphreus) morio durmitorensis **ssp. n.** (from Mt. Durmitor, northwestern Montenegro) are here described and diagnosed. The male and female genitalia and other taxonomically important characters are illustrated. The new taxa are distinctly different from the nearest relatives and represent both endemics and relicts inhabiting limited high-altitude Dinaric areas in Bosnia and Herzegovina and Montenegro. A key to *Omphreus* taxa from Montenegro and a key to subspecies of Omphreus (Omphreus) morio Dejean, 1828 are presented.

## Introduction

The genus *Omphreus* Dejean, 1828 is divided into three subgenera: *Omphreus* s. str., *Neomphreus* Winkler, 1933 and *Paromphreus* Ganglbauer, 1887. The genus is the only one constituting the tribe Omphreini (Bousquet 2003, Lorenz 2005). No phylogenetic analysis exists which makes possible to assess the true systematic position of *Omphreus* within Harpalinae. It currently contains 18 species and 13 subspecies inhabiting the Balkan Peninsula and Asia Minor (Trautner and Geigenmüller 1987, Bousquet 2003, Guéorguiev 2007, Ćurčić et al. 2008a, 2008b). All *Omphreus* taxa are distributed very locally (endemics), mostly representing montane to alpine forms living in forests (under stones and tree bark), but can be found beyond the timberline under stones as well. These ground beetles are large in size (R 16–28 mm), black colored and recognizable by the habitus and the long shaft-like antennomere 1. It is possible that the present scarcity of specimens of this genus in the collections does not mean that they are really rare, but reflects our still insufficient knowledge regarding their life history (Trautner and Geigenmüller 1987, Arndt et al. 2011).

The following *Omphreus* taxa live in Bosnia and Herzegovina (Apfelbeck 1902, 1904, Winkler 1933, Drovenik and Peks 1999, Bousquet 2003):

- Omphreus (Neomphreus) apfelbecki apfelbecki Reitter, 1893 (Loc. typ.: Bukovi peak near Bileća, Herzegovina, southern Bosnia and Herzegovina; also known from Gornje Hrasno, Hutovo Blato near Gabela, Ljubinje, Mt. Velež and Doljani, Herzegovina, southern Bosnia and Herzegovina),
- Omphreus (Neomphreus) apfelbecki cabuljensis Winkler, 1933 (Loc. typ.: Mt. Čabulja, southwestern Bosnia and Herzegovina),
- Omphreus (Neomphreus) apfelbecki dinaricus Apfelbeck, 1904 (Loc. typ.: Mt. Kamešnica, western Bosnia and Herzegovina),
- Omphreus (Neomphreus) apfelbecki plasensis Apfelbeck, 1904 (Loc. typ.: Mt. Plasa, central Bosnia and Herzegovina),
- Omphreus (Omphreus) morio morio Dejean, 1828 (Loc. typ.: Montenegro, without precise locality; also known from Mt. Orjen, southern Bosnia and Herzegovina),
- Omphreus (Omphreus) morio beckianus Ganglbauer, 1888 (Loc. typ.: Mt. Visočica, central Bosnia and Herzegovina; from central and southern Bosnia to border with Montenegro and northern Herzegovina),
- Omphreus (Omphreus) morio strupii Winkler, 1933 (Loc. typ.: Čajniče surroundings, eastern Bosnia and Herzegovina),
- Omphreus (Omphreus) weiratheri Winkler, 1933 (Loc. typ.: Mt. Prenj, southern Bosnia and Herzegovina).

The territory of Montenegro is inhabited by the following *Omphreus* taxa (Dejean 1828, Winkler 1933, Meschnigg 1934, Drovenik 1984, Drovenik and Peks 1999, Bousquet 2003, Ćurčić et al. 2008a):

- Omphreus (Neomphreus) apfelbecki meridionalis Winkler, 1933 (Loc. typ.: Kremeni Do near the village of Gornje Stravče, Mt. Žijovo, southeastern Montenegro; also known from the village of Lijeva Rijeka, Mt. Žijovo, southeastern Montenegro, and the Island of Mljet, southern Croatia),
- Omphreus (Omphreus) morio morio Dejean, 1828 [Loc. typ.: Montenegro, without precise locality; also known from the regions of Njeguši (Mt. Lovćen), southwestern Montenegro, and Krivošije (Mt. Radostak), western Montenegro],
- Omphreus (Omphreus) morio beckianus Ganglbauer, 1888 (Loc. typ.: Mt. Visočica, central Bosnia and Herzegovina; also known from northwestern Montenegro),
- Omphreus (Omphreus) morio malissorum Winkler, 1933 (Loc. typ.: Mt. Prokletije, eastern Montenegro),
- Omphreus (Omphreus) wohlberedti Winkler, 1933 (Loc. typ.: Virpazar near the Skadar Lake, southern Montenegro),
- Omphreus (Omphreus) bischoffi Meschnigg, 1934 (Loc. typ.: Mt. Mokra Gora, eastern Montenegro),
- Omphreus (Omphreus) prekornicensis Ćurčić, 2008 (Loc. typ.: Međeđe peak, village of Jugovići, Mt. Prekornica, near Nikšić, central Montenegro),
- Omphreus (Omphreus) bjelasicensis Ćurčić & Ilić, 2008 (Loc. typ.: Biogradska Gora National Park, Mt. Bjelasica, near Mojkovac, eastern Montenegro).

A few field trips organized by two of the authors of this paper (S.Ć. and D.A.) and F. Sandel and P. Zanandrea in eastern Bosnia and Herzegovina and northwestern Montenegro resulted in the discovery of the two new *Omphreus* subspecies: Omphreus (Omphreus) morio sandeli ssp. n. and Omphreus (Omphreus) morio durmitorensis ssp. n. Both the descriptions and diagnoses are presented in the current paper.

## Materials and methods

**Figures 1–3.**
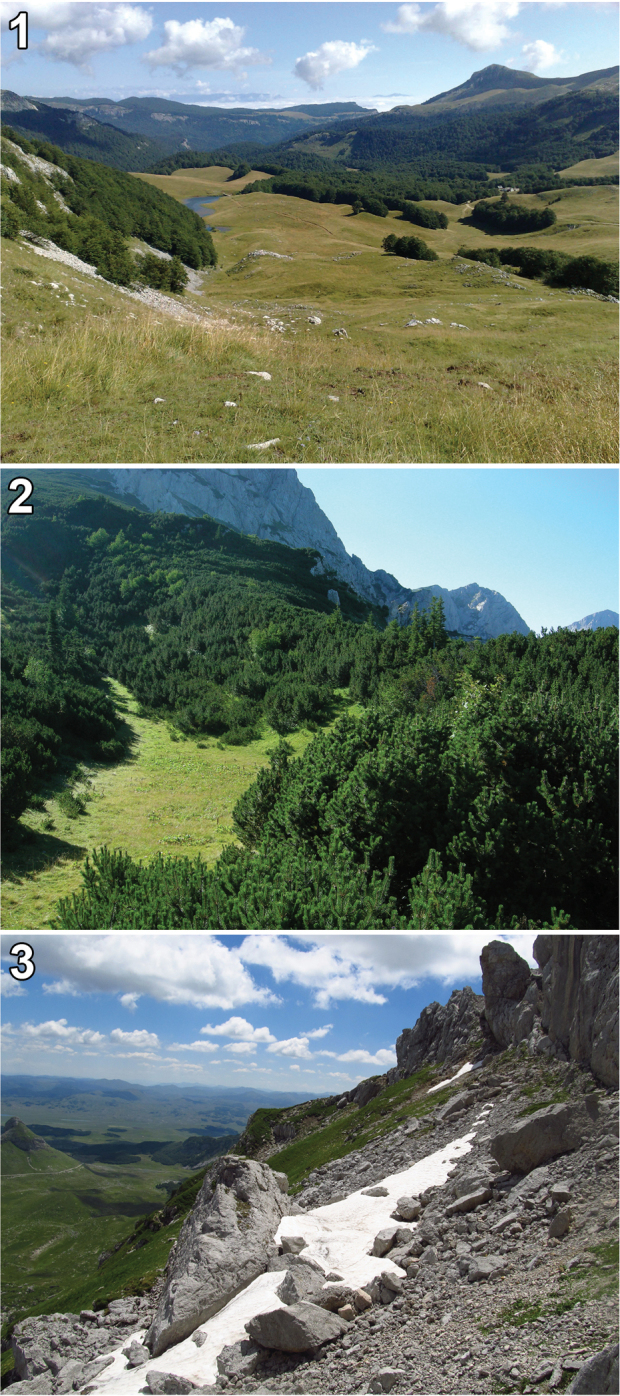
Some of the type localities of Omphreus (Omphreus) morio sandeli ssp. n. (**1, 2**) and Omphreus (Omphreus) morio durmitorensis ssp. n. (**3**). **1** eastern Bosnia and Herzegovina, southern slope of Mt. Zelengora, Čemerno, 1,450 m a.s.l., near Gacko (photo Franco Sandel) **2** eastern Bosnia and Herzegovina, Mt. Maglić, Tjentište, 1,450 m a.s.l., near Foča (photo Franco Sandel) **3** northwestern Montenegro, Mt. Durmitor, Sedlo pass, 2,100–2,200 m a.s.l., near Žabljak.

The diagnosis of Omphreus (Omphreus) morio sandeli ssp. n. is based on the study of the type series of 12 males and four females collected during 2011 and 2012 on Mt. Zelengora and Mt. Maglić (eastern Bosnia and Herzegovina), while the diagnosis of Omphreus (Omphreus) morio durmitorensis ssp. n. is based on the study of the type series of three males and two females collected during 2006, 2007 and 2014 on Mt. Durmitor (northwestern Montenegro). All specimens were collected by pitfall trapping on Mt. Zelengora, Mt. Maglić (eastern Bosnia and Herzegovina), and Mt. Durmitor (northwestern Montenegro) (Figures 1–3).

These were studied in the laboratory of the Institute of Zoology, University of Belgrade – Faculty of Biology, Belgrade, Serbia. The beetle specimens were dissected, studied, and imaged. Dry specimens and the genitalia were glued onto separate rectangular paper labels situated on the same pin.

Carl Zeiss – Stemi 2000 and Carl Zeiss Discovery V8 binocular stereomicroscopes with a Canon G10 digital camera, as well as Nikon Eclipse E100 microscope with a Moticam 2000 digital camera attached were used in the study.

### Measurements

M mean value for certain measurements

R range of the total measurements performed

TL total body length (measured from the anterior margin of clypeus to the apex of elytra)

HW/HL ratio maximum width of head/length of head

HW/PW ratio maximum width of head/maximum width of pronotum, as greatest transverse distance

AL total antennal length including the scape

AL/TL ratio total antennal length including the scape/total body length (measured from the anterior margin of clypeus to the apex of elytra)

PW/PL ratio maximum width of pronotum, as greatest transverse distance/length of pronotum (measured along the median line)

EW/EL ratio maximum width of elytra/length of elytra (as linear distance measured along the suture from the elytral base to the apex)

EL/EW ratio length of elytra (as linear distance measured along the suture from the elytral base to the apex)/maximum width of elytra

EW maximum width of elytra

### Collections

IZFB collection of the Institute of Zoology, University of Belgrade - Faculty of Biology, Belgrade, Serbia

CRS private collection of Riccardo Sciaky, Milan, Italy

CNI private collection of Nastas Ilić, Belgrade, Serbia

### Other examined taxa

Omphreus (Omphreus) prekornicensis Ćurčić, 2008: holotype male, Montenegro, Mt. Prekornica, village of Jugovići, Međeđe peak, 900 m a.s.l., near Nikšić, 28.VIII–09.IX.2002, leg. Z. Zlatić (IZFB); eight paratype males and one paratype female, same locality as for holotype, 10–28.VII.2001, leg. N. Ilić (IZFB, CNI).

Omphreus (Omphreus) bjelasicensis Ćurčić & Ilić, 2008: holotype male, six paratype males and two paratype females, Montenegro, Mt. Bjelasica (Biogradska Gora National Park), near Mojkovac, VIII.2002, leg. N. Ilić (IZFB, CNI).

Omphreus (Omphreus) ovcarensis Ćurčić & Ilić, 2008: holotype male, four paratype males and two paratype females, Republic of Serbia, Čačak, Mt. Ovčar, village of Ovčar Banja, vicinity of the Preobraženje Monastery, 24.V.1996, leg. N. Ilić (IZFB, CNI).

Omphreus (Omphreus) serbooccidentalis Ćurčić, 2008: holotype male, two paratype males and one paratype female, Republic of Serbia, Valjevo, Mt. Maljen, village of Mrčići, Bukovi peak, 900 m a.s.l., 01.VI.1997, leg. N. Ilić (IZFB, CNI).

Omphreus (Omphreus) morio serbicus Winkler, 1933: topotype male, Republic of Serbia, Mt. Murtenica, village of Draglica, 1,100 m a.s.l., 31.VII.1996, leg. N. Ilić (IZFB).

## Taxonomy

### *Omphreus* Dejean, 1828

#### Omphreus (Omphreus) morio sandeli

Taxon classificationAnimaliaColeopteraCarabidae

Ćurčić & Sciaky ssp. n.

http://zoobank.org/D7777A72-0E69-4CAC-84BC-1EAE851BD480

Figures 4–8

##### Material examined.

Holotype male labeled as follows: “eastern Bosnia and Herzegovina, southern slope of Mt. Zelengora, Čemerno, 1,450 m a.s.l., near Gacko, 22.VI–14.VII.2012, from pitfall traps, leg. F. Sandel” (white label, printed) / Holotypus Omphreus (Omphreus) morio sandeli ssp. n. S. Ćurčić & R. Sciaky det. 2014” (red label, printed) (IZFB). Paratypes: four males and one female, same data as for holotype (CRS); one male labeled as follows: “eastern Bosnia and Herzegovina, southern slope of Mt. Zelengora, Čemerno, 1,600 m a.s.l., near Foča, 07–24.VIII.2011, from pitfall traps, leg. F. Sandel” (CRS); four males and two females labeled as follows: “eastern Bosnia and Herzegovina, Mt. Maglić, Tjentište, 1,450 m a.s.l., near Foča, 23.VI–13.VII.2012, from pitfall traps, leg. F. Sandel” (CRS); two males and one female labeled as follows: “eastern Bosnia and Herzegovina, northern slope of Mt. Zelengora, Tjentište, 1,450 m a.s.l., near Foča, 23.VI–13.VII.2012, from pitfall traps, leg. F. Sandel” (CRS). All paratypes are labeled with white, printed locality labels and with red printed labels “Paratypus Omphreus (Omphreus) morio sandeli ssp. n. S. Ćurčić & R. Sciaky det. 2014”.

##### Description.

**Figures 4–8.**
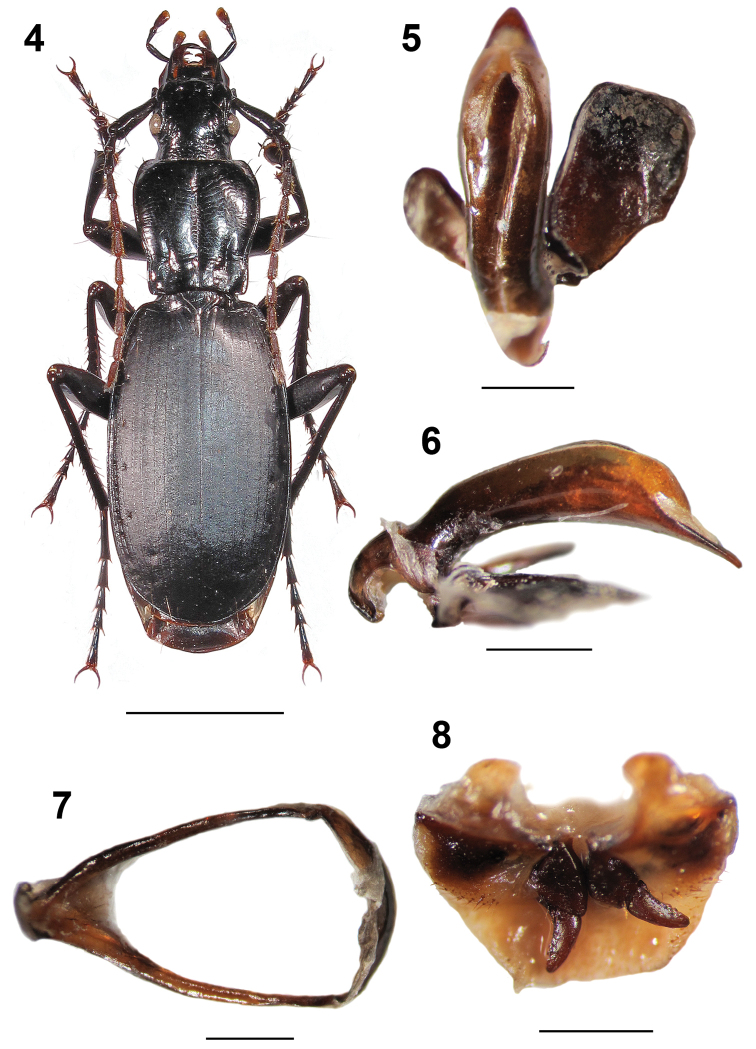
Omphreus (Omphreus) morio sandeli ssp. n. from eastern Bosnia and Herzegovina, southern slope of Mt. Zelengora, Čemerno, 1,450 m a.s.l., near Gacko. **4** holotype male, habitus (dorsal view) **5** holotype male, aedeagus (dorsal view) **6** holotype male, aedeagus (lateral view) **7** holotype male, abdominal sternite IX (urite) **8** paratype female, genitalia. Scale bars 5 mm (**4**) and 1 mm (**5–8**).

Size large: TL: R 16.88–19.73 mm (M 18.49 mm). Body elongate; elytra ovoid (Figure 4). Body color black, mouthparts, apical antennomeres, and tarsi black-brownish. Tegument shiny, except slightly matt elytra.

Head rounded, somewhat elongated [HW/HL: M 0.96 (R 0.91–1.05)], narrower (HW/PW: M 0.69) than pronotum (Figure 4). Head below eye level somewhat constricted. Labrum broad, medially rounded, carrying four setae. Epistome large, concave anteriorly, with two setae. Both vertex and occiput wrinkled. Frontal foveae deepened and long. Gula bisetose. Mandibles elongated, sickle-shaped, broadened basally. Labial palpomere 1 short, without setae. The labial palpomeres 2 and 3 longer. Both labial and maxillar palpomere 3 broadened distally and densely pubescent. AL: M 10.41 mm (R 9.25–11.16 mm). Antennae pubescent from antennomere 4. AL/TL: M 0.56 (R 0.51–0.58). Antennomere 1 club-like, sharply widened distally, with a few long setae distally, somewhat shorter than the following three antennomeres combined. Antennomere 2 slightly shorter than antennomere 3.

Pronotum sub-campaniform, elongate, PW/PL: M 0.91 (R 0.885–0.94). Fore angles somewhat prominent, rounded, hind angles obtuse, well rounded (Figure 4). Lateral margins well developed, thickened, arcuate anteriorly, then sinuate and narrowing posteriorly. Anterior pronotal margin somewhat concave, base strongly concave. Pronotum widest between its fore fourth and third. Lateral furrows narrow and shallow, with four anterior setae, one median seta, and one posterior seta each. Median furrow long and deep. Basal foveae deep and long, slightly shorter than one half of pronotum length. Pronotal disc somewhat convex proximally.

Elytra ovoid, relatively wide, rounded laterally, EW/EL: M 0.58 (R 0.54–0.61) (Figure 4). EL/EW: M 1.73. EW: M 6.26 mm in males, while 6.36 mm in females. Elytral striae shallow, weakly punctate, points somewhat deeper basally. Elytral intervals flattened. Scutellum large, triangular. Scutellar stria present, but without scutellar puncture. Elytra widest around at the middle. A setiferous puncture in interval 7 basally (close to stria 6), 4–6 setiferous punctures in interval 7 medially (close to stria 7), and two setiferous punctures situated on stria 7 apically on each elytron. The punctures deep and large. Shoulders rounded. Umbilicate series regular, with the setae densely distributed. Elytral disc somewhat convex distally.

Protarsomeres 1 and 2 widened in males. Metacoxae long and rounded. Tarsal claws elongate, glabrous, smooth (Figure 4).

Aedeagus long, median lobe somewhat widened sub-apically in dorsal view (Figure 5), while curved and strongly widened sub-apically in lateral view (Figure 6), with a straight long acute triangular apex (Figures 5 and 6). Parameres wide, the right being much broader (Figure 5). Basal bulb wide and short (Figures 5 and 6).

Male abdominal sternite IX (urite) large, sub-triangular (Figure 7).

Both gonocoxites and gonosubcoxites IX as presented in Figure 8. Gonocoxites IX wide and elongated, somewhat curved, gradually narrowing distally, each with a rounded apex, basally joined with massive rounded gonosubcoxites IX.

##### Variability.

It was noticed that the specimens from the population from the southern slope of Mt. Zelengora are of somewhat larger size and more elongate elytra (EW/EL: M 0.56) compared with the specimens belonging to the populations both from Mt. Maglić and the northern slope of Mt. Zelengora (EW/EL: M 0.59). Other characteristics, including the structure of aedeagus, do not show any significant differences among the three analyzed populations of the taxon.

##### Differential diagnosis and remarks.

The new subspecies is compared here with the morphologically nearest subspecies of Omphreus (Omphreus) morio Dejean, 1828. These are Omphreus (Omphreus) morio beckianus Ganglbauer, 1888, Omphreus (Omphreus) morio serbicus Winkler, 1933, and Omphreus (Omphreus) morio durmitorensis ssp. n. (the first antennomere being club-like distally, somewhat shorter than the following three antennomeres combined in all the subspecies mentioned) (Ganglbauer 1888, Winkler 1933).

Omphreus (Omphreus) morio sandeli ssp. n. differs from Omphreus (Omphreus) morio beckianus in the shape of the elytra (distinctly rounded laterally *vs.* slightly rounded laterally), the elytra length/width ratio (M 1.73 *vs.* around 1.66), and the body length (R 16.88–19.73 mm *vs.* 16–18 mm) (Ganglbauer 1888, Winkler 1933). The former taxon is distributed both on Mt. Zelengora and Mt. Maglić, while the latter one inhabits Mt. Visočica (the type locality) (Ganglbauer 1888). In the literature Omphreus (Omphreus) morio beckianus is reported to have a wide distribution in Bosnia and Herzegovina, Montenegro and Serbia (from central and southern Bosnia to border with Montenegro, southwestern Serbia and northern Herzegovina, including the high-altitude area of the surroundings of Sarajevo, Mts. Ivan, Bjelašnica, Volujak and Durmitor and the Lim River Valley) (Apfelbeck 1894, 1904, Wohlberedt-Triebes 1909, Drovenik 1984). This appears to us very doubtful since *Omphreus* taxa (species or subspecies) mostly inhabit single mountain ranges (Winkler 1933). The specimens from Mt. Durmitor belong to a new subspecies described herein – Omphreus (Omphreus) morio durmitorensis ssp. n.; Mt. Volujak is relatively close to Mt. Zelengora and Mt. Maglić and there is a possibility that the population from this mountain might actually belong to Omphreus (Omphreus) morio sandeli ssp. n., but this has to be proven. The specimens from all known sites should be compared in the future (including the structure of the male genitalia) in order to conclude whether they belong to a single taxon (subspecies) or to different taxa. Additionally, a detailed study of all Omphreus (Omphreus) morio subspecies (with comparisons of numerous morphological characteristics) would be needed in order to define their real taxonomic status (subspecies or species).

Omphreus (Omphreus) morio sandeli ssp. n. differs from Omphreus (Omphreus) morio serbicus in the shape of the elytra (elongate *vs.* short), the elytra length/width ratio (M 1.73 *vs.* around 1.50), and the body length (R 16.88–19.73 mm *vs.* 15–16 mm) (Winkler 1933). Omphreus (Omphreus) morio sandeli ssp. n. is found in eastern Bosnia and Herzegovina (Mt. Zelengora and Mt. Maglić), while Omphreus (Omphreus) morio serbicus lives in southwestern Serbia (Mt. Murtenica and Mt. Zlatibor) (Winkler 1933).

Eventually, Omphreus (Omphreus) morio sandeli ssp. n. differs from Omphreus (Omphreus) morio durmitorensis ssp. n. in the antennal length (M 10.41 mm *vs.* 10.38 mm), the shape of the hind pronotal angles (more rounded *vs.* less rounded), maximum width of the pronotum (between its fore fourth and third *vs.* in front of the fore third), the shape of the elytra (somewhat widened *vs.* more elongate), the elytral width/length ratio (M 0.58 *vs.* 0.56), the elytra length/width ratio (M 1.73 *vs.* 1.79), the elytral width (M 6.26 mm in males, 6.36 mm in females *vs.* 5.94 mm in males, 6.12 mm in females), maximum width of the elytra (around at the middle *vs.* slightly below the middle), the shape of the shoulders (rounded *vs.* obtusely rounded), the form of the median lobe (somewhat widened sub-apically in dorsal view, while curved and strongly widened sub-apically in lateral view, with a straight long acute triangular apex *vs.* strongly widened sub-apically in dorsal view, while arcuate, moderately widened and with a shallow concavity in the sub-apical part in lateral view, with a straight short rounded triangular apex), the shape of the basal bulb (wide and short *vs.* narrow and elongated), the shape of the male abdominal sternite IX (urite) (less elongate *vs.* more elongate), the form of the apex of the gonocoxites IX (rounded *vs.* pointed), and the total body length (M 18.49 mm *vs.* 18.13 mm).

##### Etymology.

This new subspecies is named after Franco Sandel, friend of the second author and excellent collector, who collected the whole type series of this new subspecies allowing us to freely study the material.

##### Distribution.

So far known only from the type locality (Mt. Zelengora) and the nearby Mt. Maglić, eastern Bosnia and Herzegovina.

##### Habitat.

The subspecies prefers high-altitude habitats (1,450–1,600 m a.s.l.) on Mt. Zelengora (at both the northern and southern slopes) and Mt. Maglić in eastern Bosnia and Herzegovina. Type series of the new subspecies was collected by pitfall traps filled with alcoholic vinegar, placed at different sites in beech forests by the alpine meadows on both mountains, and on a pass and near the first hairpin bend after the pass on the southern slope of Mt. Zelengora.

#### Omphreus (Omphreus) morio durmitorensis

Taxon classificationAnimaliaColeopteraCarabidae

Ćurčić & Sciaky ssp. n.

http://zoobank.org/BA83B21E-917D-4674-ACC1-B40D65FE121E

Figures 9–13

##### Material examined.

Holotype male labeled as follows: “northwestern Montenegro, Mt. Durmitor, Sedlo pass, 2,100–2,200 m a.s.l., near Žabljak, 28.VI–17.VII.2014, from pitfall traps, leg. S. Ćurčić” (white label, printed) / Holotypus Omphreus (Omphreus) morio durmitorensis ssp. n. S. Ćurčić & R. Sciaky det. 2014” (red label, printed) (IZFB). Paratypes: one female, same data as for holotype (IZFB); one male labeled as follows: “northwestern Montenegro, Mt. Durmitor, Sedlo pass, 2,100 m a.s.l., 28–29.VI.2014, from pitfall traps, leg. S. Ćurčić & D. Antić” (IZFB); one male labeled as follows: “northwestern Montenegro, Mt. Durmitor, Žabljak, 1,950 m a.s.l., VIII.2006, leg. P. Zanandrea” (CRS); one female labeled as follows: “northwestern Montenegro, Mt. Durmitor, Sedlo pass, 2,200 m a.s.l., 07.VIII.2007, leg. F. Sandel” (CRS). All paratypes are labeled with white, printed locality labels and with red printed labels “Paratypus Omphreus (Omphreus) morio durmitorensis ssp. n. S. Ćurčić & R. Sciaky det. 2014”.

##### Description.

**Figures 9–13.**
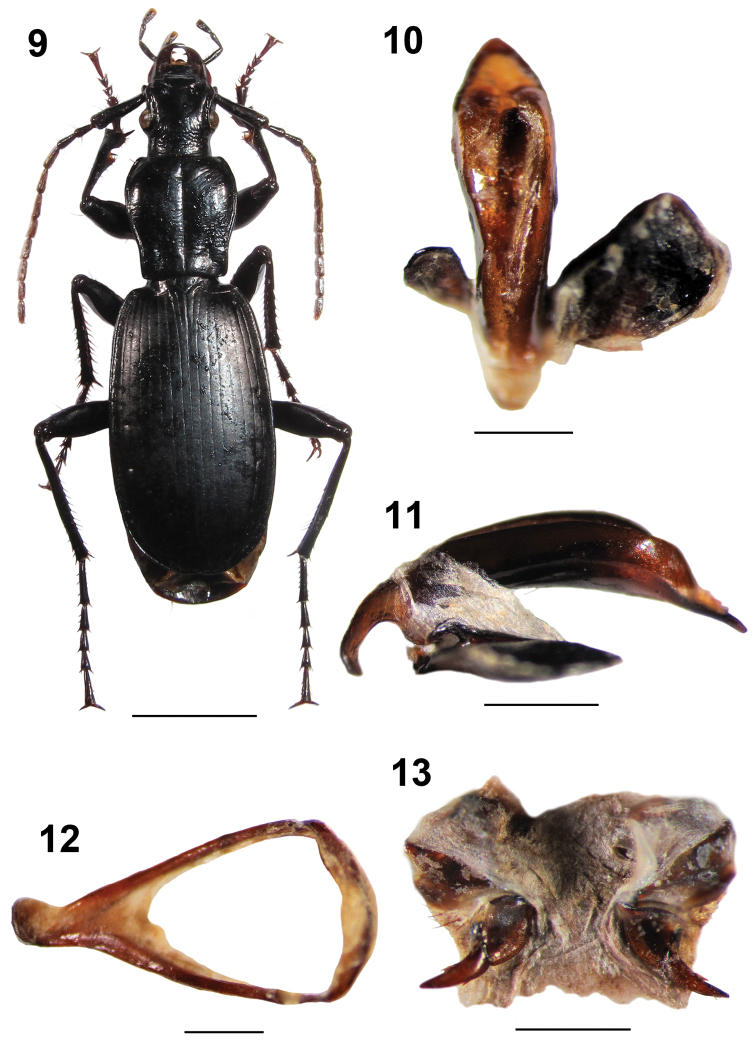
Omphreus (Omphreus) morio durmitorensis ssp. n. from northwestern Montenegro, Mt. Durmitor, Sedlo pass, 2,100–2,200 m a.s.l., near Žabljak. **9** holotype male, habitus (dorsal view) **10** holotype male, aedeagus (dorsal view) **11** holotype male, aedeagus (lateral view) **12** holotype male, abdominal sternite IX (urite) **13** paratype female, genitalia. Scale bars 5 mm (**9**) and 1 mm (**10–13**).

Size large: TL: R 17.73–18.91 mm (M 18.13 mm). Body elongate; elytra ovate (Figure 9). Body color black, mouthparts, apical antennomeres, and tarsi black-brownish. Tegument shiny, except slightly matt elytra.

Head rounded, somewhat elongated [HW/HL: M 0.95 (R 0.93–0.99)], shorter and narrower (HW/PW: M 0.695) than pronotum (Figure 9). Head beyond eye level somewhat constricted. Labrum broad, medially rounded, carrying four setae. Epistome huge, concave anteriorly, with two setae. Both vertex and occiput wrinkled. Frontal foveae deepened and long. Gula bisetose. Mandibles elongated, sickle-formed, broadened basally. Labial palpomere 1 short, without setae. Labial palpomeres 2 and 3 longer. Both the labial palpomere 3 and maxillar palpomere 3 broadened distally and densely pubescent. AL: M 10.38 mm (R 10.07–10.64 mm). Antennae pubescent from antennomere 4. AL/TL: M 0.57 (R 0.54–0.60). Antennomere 1 club-like, sharply widened distally, with a few long setae distally, somewhat shorter than the following three antennomeres combined. Antennomere 2 slightly shorter than antennomere 3.

Pronotum sub-campaniform, elongate, PW/PL: M 0.91 (R 0.90–0.92). Fore angles somewhat prominent, rounded, hind angles obtuse, somewhat rounded (Figure 9). Lateral margins well developed, thickened, arcuate anteriorly, then sinuate and narrowing posteriorly. Anterior pronotal margin somewhat concave, while the base strongly concave. Pronotum widest in front of the fore third. Lateral furrows narrow and shallow, with four anterior setae, one median seta, and one posterior seta each. Median furrow long and deep. Basal foveae deep and long, slightly shorter than half of pronotum length. Pronotal disc somewhat convex proximally.

Elytra ovate, relatively long, arcuate laterally, EW/EL: M 0.56 (R 0.53–0.58) (Figure 9). EL/EW: M 1.79. EW: M 5.94 mm in males, 6.12 mm in females. Elytral striae shallow, weakly punctate, points somewhat deeper basally; elytral intervals flattened. Scutellum large, triangular; scutellar stria present, but without scutellar punctures. Elytra widest slightly below the middle; one setiferous puncture in interval 7 basally (close to stria 6), four or five setiferous punctures in interval 7 medially (close to stria 7), and two setiferous punctures situated on stria 7 apically on each elytron; the punctures deep and large. Shoulders obtusely rounded. Umbilicate series regular, with the setae densely distributed. Elytral disc somewhat convex distally.

Protarsomeres 1 and 2 widened in males. Metacoxae long and rounded. Tarsal claws elongated, glabrous, without teeth (Figure 9).

Aedeagus long, median lobe strongly widened sub-apically in dorsal view (Figure 10), while arcuate, moderately widened and with a shallow concavity in the sub-apical part in lateral view (Figure 11), with a straight short rounded triangular apex (Figures 10 and 11). Parameres wide, the right being much huger (Figure 10). Basal bulb narrow and elongated (Figures 10 and 11).

Male abdominal sternite IX (urite) large, sub-triangular (Figure 12).

Both gonocoxites and gonosubcoxites IX as presented in Figure 13. Gonocoxites IX wide and elongated, somewhat curved, gradually narrowing distally, each with a pointed apex, basally joined with massive rounded gonosubcoxites IX.

##### Differential diagnosis and remarks.

The new subspecies is compared here with the morphologically nearest subspecies of Omphreus (Omphreus) morio. These are Omphreus (Omphreus) morio beckianus, Omphreus (Omphreus) morio serbicus, and Omphreus (Omphreus) morio sandeli ssp. n. In all these subspecies the first antennomere is club-like distally, somewhat shorter than the following three antennomeres combined (Ganglbauer 1888, Winkler 1933).

Omphreus (Omphreus) morio durmitorensis ssp. n. differs from Omphreus (Omphreus) morio beckianus in the shape of the elytra (arcuate laterally *vs.* somewhat rounded laterally), the elytra length/width ratio (M 1.79 *vs.* around 1.66), and the body length (R 17.73–18.91 mm *vs.* 16–18 mm) (Ganglbauer 1888, Winkler 1933). The former taxon is distributed on Mt. Durmitor (northwestern Montenegro), while the latter inhabits Mt. Visočica (central Bosnia and Herzegovina) (the type locality) (Ganglbauer 1888). Apfelbeck (1904) and Drovenik (1984) recorded the presence of an *Omphreus* on Mt. Durmitor reported as Omphreus (Omphreus) morio beckianus. While the former author recorded it for the mountain without precise localities, the latter author found it in several different sites (Crno Jezero Lake surroundings, forests below Mali Štulac, Zminje Jezero Lake surroundings, Veliki Štulac, Ališnica, Međed, Velika Karlica, Savin Kuk, Lokvice, Bobotov Kuk, Dobri Do, Sedlo, Todorov Do, Prutaš, Škrčka Jezera Lakes surroundings, Bolj, forest below Ćurevac, Nagorje, and Šljeme) (Apfelbeck 1904, Drovenik 1984). On the basis of the material collected from Mt. Durmitor (at Sedlo and Žabljak sites) loaned by a few colleagues, as well as the one collected by the first and third author (S.Ć. and D.A.), we have concluded that the taxon from Mt. Durmitor actually represents a new subspecies, Omphreus (Omphreus) morio durmitorensis ssp. n.

Omphreus (Omphreus) morio durmitorensis ssp. n. differs from Omphreus (Omphreus) morio serbicus in the shape of the elytra (elongate, arcuate laterally *vs.* short, rounded laterally), the elytra length/width ratio (M 1.79 *vs.* around 1.50), and the body length (R 17.73–18.91 mm *vs.* 15–16 mm) (Winkler 1933). Furthermore, they inhabit quite distant areas: Omphreus (Omphreus) morio durmitorensis ssp. n. is found in northwestern Montenegro (Mt. Durmitor), while Omphreus (Omphreus) morio serbicus lives in southwestern Serbia (Mt. Murtenica and Mt. Zlatibor) (Winkler 1933).

The diagnostic differences between Omphreus (Omphreus) morio durmitorensis ssp. n. and Omphreus (Omphreus) morio sandeli ssp. n. are presented in the differential diagnosis of the latter.

##### Etymology.

The new subspecies is named after Mt. Durmitor, its *terra typica*.

##### Distribution.

So far known only from the type locality, Mt. Durmitor, northwestern Montenegro.

##### Habitat.

The subspecies prefers high-altitude habitats (1,950–2,200 m a.s.l.) on Mt. Durmitor in northwestern Montenegro. The type series of the new subspecies was collected by pitfall traps filled with alcoholic vinegar, placed at different sites on the border between alpine meadows and rocks, up to a timberline on Mt. Durmitor.

### Key to *Omphreus* taxa from Montenegro (modified after Winkler 1933 and Ćurčić et al. 2008b)

**Table:** 

1	The first two male protarsomeres broadened. A triangular field on the top of the underside of the first male protarsomere, while the second male protarsomere on the underside almost entirely brush-like setose. The second male protarsomere square-formed (subgenus *Omphreus* s. str.)	**2**
–	The first two male protarsomeres neither broadened, nor brush-like setose on the underside. The second male protarsomere elongate. Seventh intervals of the matt elytra with 5–7 setiferous punctures each. Head behind eyes clearly narrowed. The first antennomere equally broadened towards the top, as long as the following three antennomeres combined (subgenus *Neomphreus* Winkler, 1933). Elytra elongately oval, laterally less rounded, twice as long as broad, with strongly impressed striae. Pronotum weakly heart-shaped, laterally very slightly rounded, for one fourth longer than broad. Size R 26 mm (southeastern Montenegro and southern Croatia)	**Omphreus (Neomphreus) apfelbecki meridionalis Winkler, 1933**
2	Elytra without rows of punctures in the second intervals	**3**
–	Elytra in the middle of the second intervals with a fine and dense row of punctures each, which is solely in the exterior basal and apical part absent. Head proportionally small, the first antennomere as long as the following three antennomeres combined, equally broadened towards the top. Pronotum somewhat longer than broad, in the fore third considerably strongly widened and equally rounded, the hind angles equally rounded. Elytra considerably equally oval, moderately rounded laterally, slightly more than twice as long as broad. Broadened male protarsomeres brush-like setose in a small extent. Size R 26 mm (southern Montenegro)	**Omphreus (Omphreus) wohlberedti Winkler, 1933**
3	Head well constricted behind eyes, pronotum as long as broad	**Omphreus (Omphreus) bischoffi Meschnigg, 1934**
–	Head moderately constricted behind eyes, pronotum longer than broad	**4**
4	Lateral pronotal margins narrowed or almost parallel basally, never divergent	**5**
–	Lateral pronotal margins divergent basally	**Omphreus (Omphreus) bjelasicensis Ćurčić & Ilić, 2008**
5	Lateral pronotal margins sub-parallel basally, size larger (more than 20 mm), elytral striae less impressed, elytra broader	**Omphreus (Omphreus) prekornicensis Ćurčić, 2008**
–	Lateral pronotal margins somewhat narrowed or almost parallel basally, size smaller (R 15–20 mm), elytral striae more impressed, elytra narrower [subspecies of Omphreus (Omphreus) morio Dejean, 1828]	**6**
6	First antennomere club-like thickened distally, mostly somewhat shorter than the following three antennomeres combined (northwestern Montenegro)	**Omphreus (Omphreus) morio durmitorensis ssp. n.**
–	First antennomere gradually thickened distally, as long as the following three antennomeres combined	**7**
7	Size larger (R 20 mm), pronotum anteriorly stronger widened, head proportionally smaller, with longer antennae (western and southwestern Montenegro and southern Bosnia and Herzegovina)	**Omphreus (Omphreus) morio morio Dejean, 1828**
–	Size smaller (R 17–18 mm), narrower, pronotum less widened, head proportionally larger, with shorter antennae (northern Albania and eastern Montenegro)	**Omphreus (Omphreus) morio malissorum Winkler, 1933**

### Key to the subspecies of Omphreus (Omphreus) morio (modified after Winkler 1933) (Figure 14)

**Table:** 

1	First antennomere club-like thickened distally, mostly somewhat shorter than the following three antennomeres combined	**2**
–	First antennomere gradually thickened distally, as long as the following three antennomeres combined	**5**
2	Smaller subspecies (R 15–16 mm), with elytra short (length/width ratio: M around 1.5) and well rounded laterally (southwestern Serbia)	**Omphreus (Omphreus) morio serbicus Winkler, 1933**
–	Larger subspecies (size larger than 16 mm), with elytra more elongate and less rounded laterally	**3**
3	Subspecies of somewhat smaller size (R 16–18 mm), with less elongate elytra (length/width ratio: M around 1.66) (central Bosnia and Herzegovina)	**Omphreus (Omphreus) morio beckianus Ganglbauer, 1888**
–	Subspecies of somewhat larger size (M more than 18 mm), with more elongate elytra (the length/width ratio: M > 1.66)	**4**
4	Antennae longer, hind pronotal angles more rounded, maximum width of pronotum between its fore fourth and third, elytra somewhat less elongate (length/width ratio: M 1.73) and wider, maximum width of elytra around the middle, shoulders rounded. Median lobe of aedeagus somewhat widened sub-apically in dorsal view, while curved and strongly widened sub-apically in lateral view, with a straight long acute triangular apex, basal bulb wide and short, male abdominal sternite IX (urite) less elongate, apex of the gonocoxites IX rounded, somewhat larger body size (M 18.49 mm) (eastern Bosnia and Herzegovina)	**Omphreus (Omphreus) morio sandeli ssp. n.**
–	Antennae shorter, hind pronotal angles less rounded, maximum width of pronotum in front of the fore third, elytra more elongate (length/width ratio: M 1.79) and narrower, maximum width of elytra slightly below the middle, shoulders obtusely rounded. Median lobe of aedeagus strongly widened sub-apically in dorsal view, while arcuate, moderately widened and with a shallow concavity in the sub-apical part in lateral view, with a straight short rounded triangular apex, basal bulb narrow and elongated, male abdominal sternite IX (urite) more elongate, apex of the gonocoxites IX pointed, somewhat smaller body length (M 18.13 mm) (northwestern Montenegro)	**Omphreus (Omphreus) morio durmitorensis ssp. n.**
5	Elytra short, around 1 and ½ times longer than broad, evidently widened laterally, length R 18–19 mm (eastern Bosnia and Herzegovina)	**Omphreus (Omphreus) morio strupii Winkler, 1933**
–	Elytra slender, at least for 1 and 2/3 times longer than broad, less rounded and widened laterally	**6**
6	Pronotum markedly longer than broad, somewhat narrowed towards the hind angles, elytra slender, almost twice as long as broad, with weakly expressed shoulders	**7**
–	Pronotum slightly longer than broad, with sides basally parallel, the hind angles less rounded, elytra around 1 and 2/3 times longer than broad, hardly rounded laterally, with strongly expressed shoulders, length R 18–19 mm (northern, northeastern and central Albania and southern Serbia)	**Omphreus (Omphreus) morio albanicus Apfelbeck, 1906**
7	Size larger (R 20 mm), pronotum anteriorly stronger widened, head proportionally smaller, with longer antennae (western and southwestern Montenegro and southern Bosnia and Herzegovina)	**Omphreus (Omphreus) morio morio Dejean, 1828**
–	Size smaller (R 17–18 mm), narrower, pronotum less widened, head proportionally larger, with shorter antennae (northern Albania and eastern Montenegro)	**Omphreus (Omphreus) morio malissorum Winkler, 1933**

**Figure 14.**
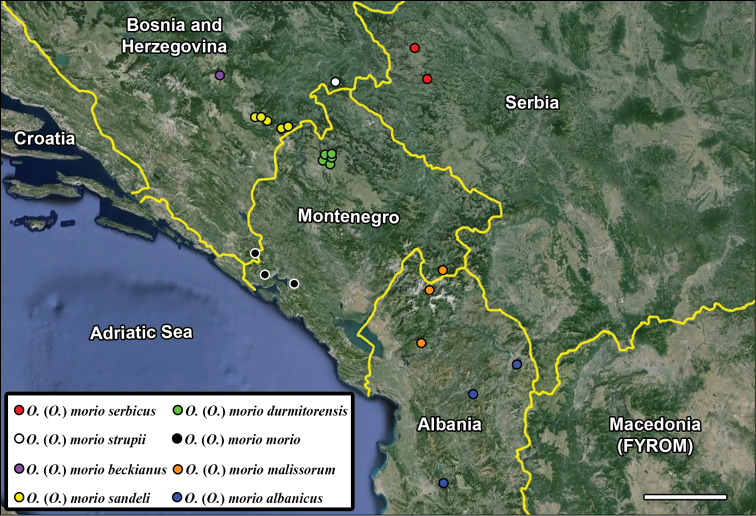
The distribution of Omphreus (Omphreus) morio subspecies. Scale bar 50 km.

## Supplementary Material

XML Treatment for Omphreus (Omphreus) morio sandeli

XML Treatment for Omphreus (Omphreus) morio durmitorensis
